# PHI-base: a new interface and further additions for the multi-species pathogen–host interactions database

**DOI:** 10.1093/nar/gkw1089

**Published:** 2016-12-03

**Authors:** Martin Urban, Alayne Cuzick, Kim Rutherford, Alistair Irvine, Helder Pedro, Rashmi Pant, Vidyendra Sadanadan, Lokanath Khamari, Santoshkumar Billal, Sagar Mohanty, Kim E. Hammond-Kosack

**Affiliations:** 1 Department of Plant Biology and Crop Science, Rothamsted Research, Harpenden, Hertfordshire AL5 2JQ, UK; 2 Cambridge Systems Biology and Department of Biochemistry, University of Cambridge, Sanger Building, 80 Tennis Court Road, Cambridge, Cambridgeshire CB2 1GA, UK; 3 The European Molecular Biology Laboratory, The European Bioinformatics Institute, Hinxton, Cambridgeshire CB10 1SD, UK; 4 Molecular Connections Private Limited, Basavanagudi, Bangalore 560 004, India

## Abstract

The pathogen–host interactions database (PHI-base) is available at www.phi-base.org. PHI-base contains expertly curated molecular and biological information on genes proven to affect the outcome of pathogen–host interactions reported in peer reviewed research articles. In addition, literature that indicates specific gene alterations that did not affect the disease interaction phenotype are curated to provide complete datasets for comparative purposes. Viruses are not included. Here we describe a revised PHI-base Version 4 data platform with improved search, filtering and extended data display functions. A PHIB-BLAST search function is provided and a link to PHI-Canto, a tool for authors to directly curate their own published data into PHI-base. The new release of PHI-base Version 4.2 (October 2016) has an increased data content containing information from 2219 manually curated references. The data provide information on 4460 genes from 264 pathogens tested on 176 hosts in 8046 interactions. Prokaryotic and eukaryotic pathogens are represented in almost equal numbers. Host species belong ∼70% to plants and 30% to other species of medical and/or environmental importance. Additional data types included into PHI-base 4 are the direct targets of pathogen effector proteins in experimental and natural host organisms. The curation problems encountered and the future directions of the PHI-base project are briefly discussed.

## THREE KEY POINTS

- Improved PHI-base 4 platform with increase in pathogen species and genotypic/phenotypic data content.
- On-line author curation tool called PHI-Canto for any pathogenic species and a PHIB-BLAST function.
- Cross-references for first plant host targets of pathogen effectors.

## INTRODUCTION

With an increasing world population and associated global trade, pathogens pose a constant threat to efforts focused on increasing crop yield, whilst safeguarding human and animal health and protecting natural ecosystems (1). Since the identification of the first bacterial avirulence gene in the plant infecting pathogen *Pseudomanas syringae* in the early 1980s (2,3), many more research articles have been published describing the molecular analysis of pathogen gene mutations via experimentation and the functional characterization of these mutant alleles on one or more host organisms.

The pathogen–host interactions database (www.phi-base.org) is a well recognized information resource cataloguing the phenotypes of experimentally verified pathogenicity, virulence and effector genes from a wide range of plant and animal pathogens and host species. The trained curation team manually extracts the information about gene function in pathogenesis from peer reviewed literature and transfers the data into a computable form. This enables immediate data access to guide further experimentation and enable comparative data analysis. Various complementary multi-species databases on pathogens exist that also provide gene function annotation. Recently, these have been comprehensively reviewed (4,5).

PHI-base data were first made available via the internet (www-phi3.phibase.org) in May 2005 (6). The data content has been continually updated (7). Since 2011, PHI-base has provided phenotypic annotation for over 100 crop plant infecting microbial pathogens into Ensembl Genomes (8) as part of the PhytoPath project (9). With the help of part-time biocurators and continued support of the phytopathogen research community ∼10 papers are curated weekly and new data releases occur twice yearly.

PHI-base was intentionally set up to cover a wide scope of microbial species and their hosts to facilitate cross-kingdom comparative phenomics, genomics and network approaches for virulence gene discovery and to identify potential disease intervention targets (10,11). The database provides outlinks to primary information sources including (i) PubMed, (ii) the digital object identifier for the curated article, (iii) NCBI taxonomy for organism details and (iv) to UniProt Knowledgebase as a resource for functional up-to-date information on proteins. Detailed comparative analyses across wide taxonomic distances can be made using nine high-level ‘species-neutral’ PHI-base phenotypes. This controlled vocabulary has been defined previously (7) and is routinely used in research publications but the individual terms have not been mapped to gene ontology (GO) terms (12) due to their generalized nature. Phenotypes are assigned to interactions (interaction phenotype) that are defined as the observable function of one gene, on one host and one tissue type. Only one term is assigned per pathogen–host interaction. These phenotypes include four terms on ‘disease causing ability’ such as ‘loss of pathogenicity’, ‘reduced virulence’ and ‘unaffected pathogenicity’ as well as ‘resistance or sensitivity to chemistry’.

Version 4.2 of PHI-base released in October 2016, and described in this article, contains information on 4460 genes, 8046 interactions, 264 pathogens, 176 hosts and 2219 references. Plant infecting pathogens (bacteria, fungi, protists and nematodes) represent 70% of the species in PHI-base. The majority of these plant pathogens are highly detrimental to the productivity of either staple agricultural and horticultural crops, for example, rice, maize, wheat, barley, rye, potato, brassica, bean, pepper, tomato and/or commercial tree species, for example, olive, apple, pear and pine. The other 30% of the pathogenic species in PHI-base are of medical or ecological importance and infect humans, farmed animals, farmed fish and shell fish, cultivated mushrooms, honey bees, wild birds and/or specific insects.

In the following we describe three major improvements to the database: a new PHI-base web platform to search the database including BLAST functions, increased data content and a newly developed multi-species annotation tool, called PHI-Canto. PHI-Canto strongly supports the community annotation of a wide repertoire of pathogen gene types required for disease formation on host species, pathogen effectors that activate or suppress plant host defenses, the novel ‘in host’ functions of variant effector sequences, effector targets in plants and the target sites of commercial anti-infectives.

## RESULTS AND DISCUSSION

### PHI-base 4: a new database search interface

The previous version of the PHI-base 3 relational database (online without major updates to the computational framework since 2005 at www-phi3.phibase.org), was able to display only a limited amount of the information currently being curated. Since PHI-base inception, curation depth was continuously increased to be able to extract, quantify and define about twice the amount of data from every research article using both generic and bespoke ontologies. Currently every article is scanned for information to be extracted into a record spreadsheet with 81 fields (Supplementary Text 1). In PHI-base 4 the majority of this information is now displayed in a faceted interface. The new information presented includes additional details on the pathogen gene modifications (such as a deletion containing a gentamicin resistance cassette), further details on pathogen and host strains, increased host data including genotype, plant resistance gene tested, tissue used in the inoculation bioassays and the first plant host target proteins physically interacting with curated pathogen effector proteins (Supplementary Table S1).

The new PHI-base 4 platform (www.phi-base.org) uses the Java programing language Version 1.7 and the Apache Lucene/SOLR NoSQL platform Version 4.7.1 (https://lucene.apache.org) to allow full text indexing and faceted searching capability. The indexing approach allows increased flexibility in search terms. Keyword searches are now made possible using text autocomplete features. In the current workflow, twice yearly the spreadsheet with curated data (downloadable from the website) is parsed into a MySQL database (version 5.0) (schema in Supplementary Figure S1) and indexed using SOLR. The web interface is generated in Apache Tomcat 6.0 using JavaServer Pages on CentOS 5.9 as a computing platform.

**Figure 1.**
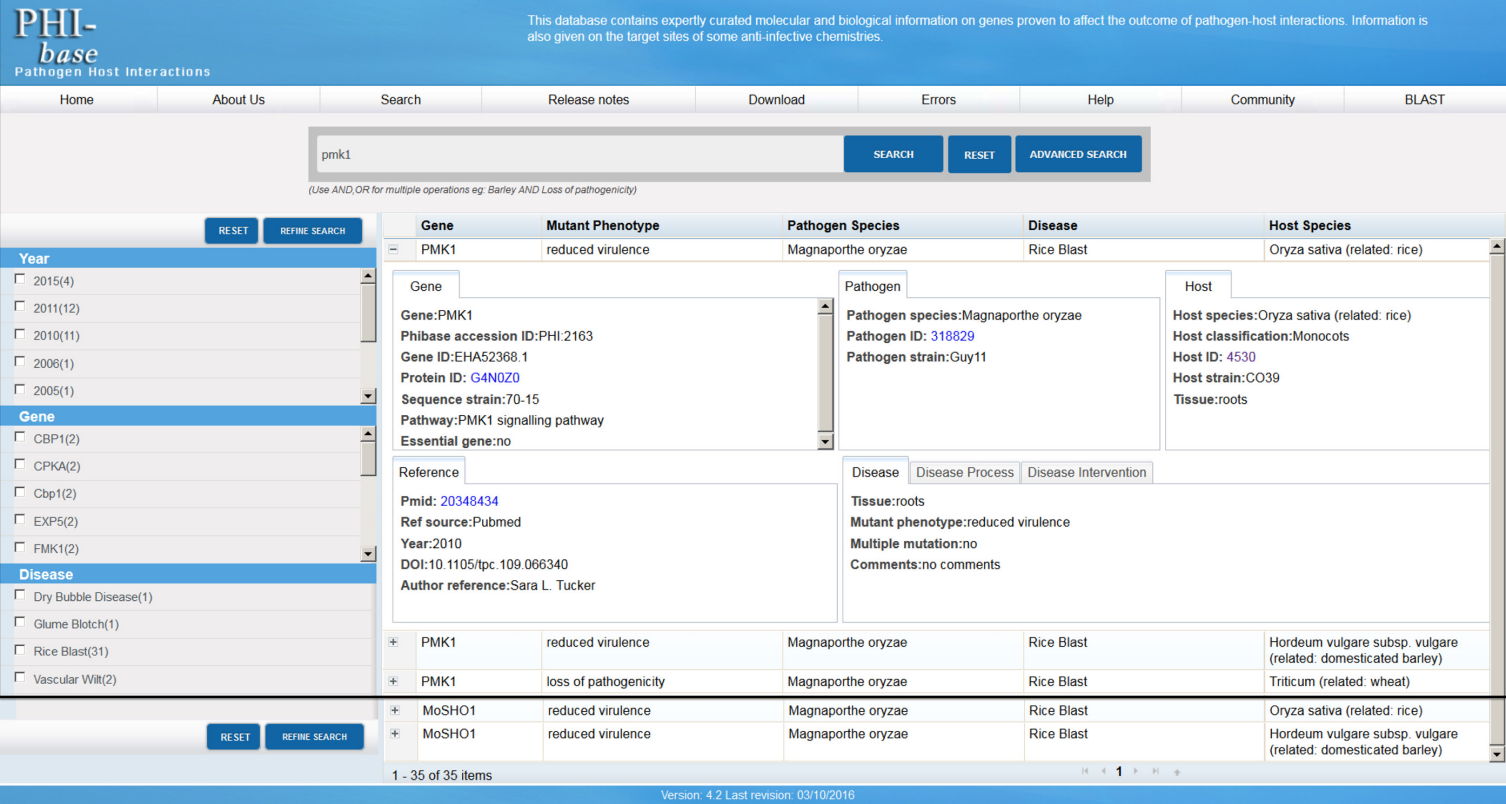
Search results for the gene *PMK1* in PHI-base version 4. Information is displayed in facets. The main page shows a detailed view for the selected record PHI:2163. Here the *Magnaporthe oryzae pmk1* mutant was tested on roots and the observed mutant phenotype was ‘reduced virulence’. All retrieved 35 records are listed on the main page. Facet views on the left provide summary results and can be used to refine search results further.

The interface displays keyword search results in faceted views. For example, search results for the ‘pmk1’ gene (Figure 1) are summarized on the left hand site giving information divided into nine summary facets including publication year, gene name, disease name, pathogen species, mutant phenotypes, experimental technique, host species and multiple mutation. In round brackets next to each summary result are displayed the number of retrieved records. The check box to the left can be used to select the records and to refine the search. The main result page panel display provides an overview of the individual retrieved records, where the search term(s) were found, displaying record information in five columns (gene name, the observed mutant phenotype, pathogen species, disease name and host species). Selecting the plus toggle button to the left of the gene name reveals detailed information on each gene record curated from one article. Information is displayed in seven sub-facets: gene, pathogen, host, reference, disease, disease process and disease intervention. A video tutorial explaining to users the PHI-base 4 search functionality is provided online (https://www.youtube.com/watch?v=5DLXo0_brMo).

## PHIB-BLAST

PHI-base is primarily a knowledge provider and bioinformatics tools to analyze PHI-base data are provided elsewhere. For example, advanced search functionality is provided by PhytoPath (www.PhytoPathdb.org) based on BioMart search functionality to enable searches for genes of interest across different species of plant pathogens provided in Ensembl Genomes (9). In the PhytoPath-Advanced-Search tool, filters can be set for specific search terms that include PHI-base interaction phenotypic outcomes, PHI-base experimental conditions and PHI-base hosts.

Feedback received from the PHI-base user community indicated that many users would appreciate the availability of a BLAST tool within PHI-base to determine whether a homolog of their ‘gene of interest’ had already been explored experimentally in a pathogenic species. Therefore, a PHIB-BLAST tool is provided to allow the direct query of the translated PHI-base protein set. Archived versions of PHI-base 4 protein sets are provided for reproducibility purposes. PHIB-BLAST functionality was established using SequenceServer Version 1.08 (www.sequenceserver.com) to provide the graphical user interface (Supplementary Figure S2). Batch BLAST of multiple protein query sequences is also supported.

### PHI-Canto: toward development of a multi-species pathogen–host interactions curation interface for authors

Biocuration of plant–host interaction experiments is complex and the curation process is difficult to automate. Extracting a complete curation dataset from publications can be challenging where data may be lacking from the primary publication content. Such issues can arise from journal restrictions on document length, authors using community specific sources for gene/protein identifiers rather than UniprotKB protein identifiers and common organism names rather than those with specific NCBI taxonomy identifiers. Authors of primary publications are the most knowledgeable persons to enter their research data into a database. However, this may require effective literature curation tools and guidance from trained biocurators.

A literature curation prototype tool has now been adapted for PHI-base, called PHI-Canto (http://curation.phi-base.org). The PHI-Canto tool is a prototype implementation of the Canto community curation tool successfully in use for the PomBase fission yeast database since 2014 (13). As in PomBase (https://curation.pombase.org) researchers are directly able to contribute annotations to PHI-base based on their publications. The web-based tool enables both curators and researchers to create annotations using a web browser. GO terms, phenotypes and interactions are supported. Additional information can be captured using annotation extensions to generate ‘on-the-fly’ terms. These post-compositional terms are initially less restrictive than pre-composed ontology terms and may lead to the future development of new pre-composed terms (14). As PHI-base is a multi-species database compared to PomBase, PHI-Canto had to be adapted to enable curation of multiple pathogen species and phenotypic outcomes of specific pathogen–host interactions. Data in PHI-Canto are captured by entering a pathogen gene and making a pathogen gene annotation. Host data are entered with the help of annotation extensions. Data requirements for PHI-Canto are: (i) data must come from a peer-reviewed publication with a PubMed ID. The predicted coding sequence of the gene(s) for curation must have (ii) a UniProtKB protein accession number(s) to create a systematic identifier and linkout to a specific organism. To prevent data duplication PHI-Canto can only have one assigned curator per PubMed ID. Each author can have several papers under curation at the same time. PHI-Canto curation guidelines (http://curation.phi-base.org/docs) have been made available based on revised help documents from PomBase-Canto. A tutorial video curating an example paper by Cuzick *et al*. (15) is available online (https://www.youtube.com/watch?v=-26pfqgWf2Q).

### Biological data

#### Phenotype data in the current release of PHI-base

PHI-base version 4.2 includes 27% more phenotype interactions compared to PHI-base version 3.8 described previously (4). Bacterial and fungal pathogens represent the majority of the interaction data (Table 1). A total of 3029 phenotype interactions describing experimental data on 898 genes taken from 407 newly curated research articles are included up to a 2016 publication date. An additional 33 pathogenic organisms have been included into PHI-base for the first time. The highest number of pathogen–host interactions tested in molecular genetics studies and reported in the literature include the filamentous fungal pathogens *Fusarium graminearum* and *Magnaporthe oryzae* causing diseases on main staple food crops such as wheat, rice and maize. For the animal kingdom the most frequently studied pathogens include the human pathogens *Candida albicans* and *Salmonella enterica* (Supplementary Table S2). To permit wide interspecies comparisons, PHI-base curates for each experimentally tested gene a high-level interaction phenotype. A comparable number of interactions is reported for most PHI-base phenotype outcomes. However, there is one exception. For the category ‘effector’, most interactions were tested in bacterial host interactions where currently effector discovery tested on host plant species is leading the field (Table 2).

**Table 1.** Summary of pathogen groups, interactions and phenotypes within PHI-base version 4.2

Phenotype/Pathogen^1^	Bacterium	Fungus	Protist	Nematode
Number of pathogens	135	110	15	4
Interactions in total	4038	3689	299	10
Loss of pathogenicity	358	326	3	1
Reduced virulence	1546	1569	47	5
Unaffected pathogenicity	656	1338	7	0
Effector (plant avirulence determinant)	1269	172	214	3
Increased virulence (hypervirulence)	187	114	22	1
Lethal	15	132	6	0
Chemistry target: resistance to chemical	5	30	0	0
Chemistry target: sensitivity to chemical	2	4	0	0
Enhanced antagonism	0	4	0	0

^1^First two rows are summary rows. All other rows list the number of interactions per PHI-base phenotype and pathogen group.

**Table 2.** Summary of the number of host species and interactions within PHI-base version 4.2

Phenotype	Plant	Vertebrate	Insect	Nematode	Other^1^
Host species	121	24	19	2	10
Interactions in total	5710	1865	277	84	32
Loss of pathogenicity	533	143	8	3	1
Reduced virulence	1763	1174	161	42	27
Unaffected pathogenicity	1639	280	62	18	2
Effector (plant avirulence determinant)	1533	111	18	5	1
Increased virulence (hypervirulence)	129	150	28	16	1
Lethal^2^	78	3	0	0	0
Chemistry target: resistance to chemical	26	3	0	0	0
Chemistry target: sensitivity to chemical	5	1	0	0	0
Enhanced antagonism	4	0	0	0	0

^1^Other hosts include organisms belonging to the taxons of fungi, Crustacea and slime mold (*Dictyostelium discoideum*).

^2^Sixty eight interactions have no specific host associated due to proposed mutant lethality *in vitro*.

Most host species in PHI-base belong to the plant kingdom (∼70%) while vertebrates species comprise 14% (Table 2 and Supplementary Table S3). It can be noted that the number of insect and other invertebrate host species (i.e. Crustaceae) is on the increase. More recent research activities into biological control for insect species as well as increased adoption of the 3Rs principle are a contributing factor. The phenotype outcome ‘increased virulence (hypervirulence)’ is currently more often observed for vertebrates than for plant hosts.

A major curation emphasis over the past two years was to increase the coverage of gene deletions which resulted in a hypervirulence phenotype on the host. This has risen from 112 genes in PHI-base version 3.8 to 233 genes (tested in 324 interactions) for PHI-base 4.2. A subset of these mutations were linked to naturally emerging infectious disease outbreaks in a recent review by Brown *et al*. (10). This gene set may warrant closer monitoring in pathogen populations when attempting to mitigate the spread of hypervirulent pathogens.

A second major emphasis of PHI-base curation was to increase the pathogen effector gene coverage. The number of effector interactions has increased from 172 in PHI-base 3.8 to 1668 interactions in PHI-base 4.2. Pathogen effectors contribute to disease in both the animal and plant kingdoms. For PHI-base a high-level interaction phenotype ‘effector (plant avirulence determinant)’ was introduced (7). In the plant kingdom, the presence, absence or modification of specific disease resistance loci and/ or disease susceptibility loci possessed by individual cultivars or ecotypes determines the interaction outcome. Here, an effector protein is required for the direct or indirect recognition of a pathogen only in host genotypes which possess the corresponding disease resistance gene or disease susceptibility gene. Positive recognition by the plant host either leads to activation of plant defenses and the pathogen fails to cause disease or leads to the activation of processes that result in disease susceptibility. However, the terminology is currently under revision in collaboration with the GO consortium and further changes that fulfill community curation needs are likely to be included in a future PHI-base release.

#### Curation of novel data types: plant host targets of pathogen effectors

To extend the scope of PHI-base data we have begun to connect data from plant pathogen effectors to the first interacting host targets (examples given in Table 3). Our aim is to introduce pathogen effector and their host target annotations directly in genome browsers as part of the PhytoPath project (9). In this approach experimentally identified interacting proteins are mapped via their corresponding transcript IDs to Ensembl Genomes. In PHI-base this style of ‘coupled’ curation is still in its infancy due to the fact that information has to be extracted from several publications with gene transcript accessions assigned often in different database formats. In the examples given, plant pathogen proteins with an ‘effector’ phenotype were mapped to their respective genomes in Ensembl Fungi or Protists and genome transcript IDs were recorded for each of these entries. Host proteins interacting with these selected pathogen proteins were identified in literature searches and relevant publications describing physical protein interactions such as yeast two hybrid, co-immunoprecipitations and bimolecular fluorescence complementation were selected. The identified first plant host target proteins were mapped in Ensembl Plants to extract the genome transcript IDs. In PHI-base the first mapped host target data is displayed under the disease facet in the results page. In these cases of ‘coupled’ curation the additional Ensembl Genomes stable IDs have been added to the the facets for pathogen gene ID and the host target ID (visible under the gene and disease results facets, respectively).

**Table 3.** New data type curation - linking pathogen effectors with their host targets

PHI-base ID	Pubmed ID	Pathogen^1^	Effector	Ensembl ID^2^	Host^3^	First host target	Ensembl plant ID	Interaction evidence^4^
PHI:2744	23459172	*Um*	Pit2	EF: KIS71480	*Zm*	CP2	GRMZM2G038636_T0.1	yeast two-hybrid; Co-IP
PHI:4251	15096512	*Pi*	EPI1	EP: PITG_22681T0	*Sl*	P69B	Solyc08g079870.1.1	Co-IP
PHI:4252	15980196	*Pi*	EPI10	EP: PITG_12129T0	*Sl*	P69B	Solyc08g079870.1.1	Co-IP
PHI:4258	25284001	*Ha*	HaRxL106	EP: HpaT814111	*At*	MOS6	AT4G02150.1	BiFC; Co-IP
PHI:4751	24339748	*Ha*	HaRxL44	EP: HpaT808319	*At*	MED19a	AT5G12230.1	BiFC; Co-IP
PHI:4253-4257^5^	20601497	*Ha*	ATR1^NdWs-B^	EP: HpaT801867	*At*	RPP1	AT3G44480.1	Co-IP

^1^*Hyaloperonospora arabidopsidis* (*Ha*), *Phytophthora infestans* (*Pi*), *Ustilago maydis* (*Um*).

^2^Ensembl Protist (EP), Ensembl Fungi (EF). Transcript IDs are listed.

^3^*Zea mays* (*Zm*), *Solanum lycopersicum* (*Sl*), *Arabidopsis thaliana* (*At*).

^4^Co-immunoprecipitation (Co-IP), Bimolecular fluorescence complementation (BiFC).

^5^Five *ATR1^NdWs^*^-B^ containing *Hyaloperonospora arabidopsidis* isolates were assessed in one publication.

### Other developments

The PHI-base version 4.2 release reported here is mapped to the Ensembl Genomes release 32 as part of the PhytoPath project (9). In an effort to encourage the wide adoption and reuse of PHI-base data, we now provide a Creative Commons Attribution-No Derivatives 4.0 International License for data downloads. Additional documents including a species list of all pathogens in PHI-base are downloadable from the website. We expect that this extra information will enable scientists not familiar with details on pathogens and their hosts to use PHI-base data in computational studies.

Major efforts are currently underway to promote data integration and interoperability of data services. PHI-base recently joined (via the UK node) the European ELIXIR project (https://www.elixir-europe.org) that provides a training and network platform for European academic service and database providers to connect their sources. Guiding principles for scientific data management and stewardship were recently set out by the ‘FAIR’ data initiative (16). Applying these principles to PHI-base, Rodriguez-Iglesias *et al.* created a plant pathogen database, a subset of PHI-base without the animal records, called ‘Semantic PHI-base’ (17). The aim of the modified database is to offer users the ability to ask questions across multiple connected databases that share common identifiers and structure.

### Community adoption and future plans

Understanding how microbial pathogens infect their hosts is of prime importance to prevent and control disease outbreaks. PHI-base has repeat users in 127 countries. The complete database content can be downloaded, permitting use by scientists for advanced bioinformatics analyses and uses in industry. To date, 131 peer reviewed publications have cited PHI-base. All articles are listed in the ‘about’ section of the website to provide database use examples. New uses for PHI-base data include (i) comparative analysis of the genomes of fungi that infect insects in natural ecosystems and alter insect behavior to favor fungal spore dispersal (18,19), (ii) characterization of the potential virulence genes within the genomes of eight dematiaceous fungal species which infect humans in tropical regions (20) or facilitate ascomycete fungal endoparasitism of nematodes (21), (iii) exploration of divergent and convergent evolution of fungal pathogenicity in insect, plant and human hosts (22) and (iv) studying the rate of horizontal gene transfer events from pathogenic bacteria to plant pathogenic fungi (23).

Data curation for PHI-base will encounter new challenges as new data types are being reported in the literature. These new data types include (i) siRNA (small interfering RNAs) of pathogen and host origin that enable bidirectional trans-kingdom gene silencing (24), (ii) provision of unique identifiers for non-proteinaceous pathogen effectors such as chitin oligomers and small metabolites and (iii) capturing molecular interaction data between pathogen and host proteins reported in crystallographic studies.

The adoption and consistent use of ontologies as annotation vocabularies is an additional important expansion area for PHI-base to allow data integration and enable phenotype searches across kingdoms (25). Ontology development for microbial pathogen–host interactions is not straightforward and there is an urgent need for further ontology development. This is demonstrated by the fact that from ∼700 terms developed over a 3-year period in the PAMGO (Plant-Associated Microbe Gene Ontology) consortium (26), 117 terms were made obsolete again by GO due to inconsistencies or scope for improvement. Thus the PHI-base biocurators are currently developing additional preferably species-neutral ontology terms in association with the PomBase team (Cambridge, UK) and species experts within the global plant-pathogen scientific community.

We welcome user feedback and suggestions of publications for inclusion in PHI-base by email to contact@phibase.org. The Chrome web browser is recommended for optimal PHI-base use.

## AVAILABILITY

1. PHI-base 4: www.phi-base.org
2. Data exchange link: http://www.phi-base.org/Downloadable/phi42_accessions.xml
3. PHI-base 4 training tutorial: https://www.youtube.com/watch?v=5DLXo0_brMo
4. PHIB-BLAST: http://phi-blast.phi-base.org
5. PHI-Canto (multi-species community annotation tool): http://curation.phi-base.org
6. PHI-Canto training video: https://www.youtube.com/watch?v=-26pfqgWf2Q
7. Previous version PHI-base 3: http://www-phi3.phibase.org/
8. Linked resource - PhytoPathdb: http://www.phytopathdb.org
9. Linked resource: http://ensemblgenomes.org
